# Short- and Medium-Term effects of major Ozone therapy on disease parameters in fibromyalgia syndrome: A retrospective study

**DOI:** 10.1007/s00296-025-05827-1

**Published:** 2025-03-12

**Authors:** Ahmet Üşen, Didem Sezgin Özcan, Mehmet Ağirman, Hilal Güner, Burhan Fatih Kocyigit

**Affiliations:** 1 Faculty of Medicine, Department of Physical Medicine and Rehabilitation, Istanbul Medipol University, Istanbul, Türkiye Turkey; 2 Medicana International Ankara Hospital, Ankara, Türkiye Turkey; 3 Kahramanmaraş Necip Fazıl City Hospital, Kahramanmaraş, Türkiye Turkey; 4 Adana City Research and Training Hospital, Adana, Türkiye Turkey; 5 TEM Avrupa Otoyolu Göztepe Çıkışı No: 1, Bagcilar, Istanbul, 34214 Turkey

**Keywords:** Fibromyalgia syndrome, Ozone therapy, Autohemotherapy

## Abstract

**Background:**

Fibromyalgia syndrome (FMS) is a chronic condition causing widespread pain, fatigue, and sleep disturbances. Conventional treatments often provide limited relief, leading to growing interest in complementary therapies like ozone therapy.

**Objective:**

This study aims to retrospectively evaluate the short- and medium-term efficacy of ozone therapy in patients with FMS, focusing on changes in pain, functional status, sleep quality, fatigue, anxiety, and depression.

**Methods:**

Twenty-five FMS patients treated at the Physical Medicine and Rehabilitation outpatient clinic of University Hospital were included. Participants underwent a 10-session major ozone autohemotherapy protocol administered twice weekly. Key outcomes were measured using the Visual Analog Scale (VAS), Fibromyalgia Impact Questionnaire (FIQ), Hospital Anxiety and Depression Scale (HADS), Pittsburgh Sleep Quality Index (PSQI), and Fatigue Severity Scale (FSS) at baseline, post-treatment, and six months post-treatment. Generalized Estimating Equations were used for data analysis.

**Results:**

VAS scores decreased from 6.4 to 3.68 post-treatment (*p* < 0.001) and partially increased to 4.12 at six months (*p* = 0.01). Similar trends were observed for FIQ, HADS, PSQI, and FSS. Tender points declined from 14.36 to 9.8 post-treatment (*p* < 0.001) and remained stable at 10.12 at six months (*p* = 0.289). FIQ scores improved from 59.2 to 39.08 post-treatment (*p* < 0.001) and stabilized at 40.12 at six months (*p* = 0.328).

**Conclusion:**

Ozone therapy demonstrates promising short- and medium-term efficacy in managing FMS symptoms, with significant improvements post-treatment. However, partial symptom recurrence at six months suggests the need for optimized protocols and further studies to ensure long-term sustainability.

**Supplementary Information:**

The online version contains supplementary material available at 10.1007/s00296-025-05827-1.

## Introduction

Fibromyalgia Syndrome (FMS) is a complex and chronic disorder primarily characterized by chronic widespread musculoskeletal pain, accompanied by fatigue, sleep disturbances, cognitive dysfunction and emotional distress. The prevalence of FMS in the general population is estimated to be 2–4%, with a higher frequency observed in women compared to men [1]. Although the etiology of FMS is not fully understood, it is thought to involve an interplay of genetic predisposition, environmental triggers, neuroendocrine dysfunctions and central sensitization mechanisms [2, 3]. In FMS, widespread musculoskeletal pain is often accompanied by symptoms such as stiffness, tenderness, concentration difficulties, memory problems, anxiety, and depression. This condition significantly impairs patients’ ability to perform daily tasks and maintain social and professional responsibilities, leading to a diminished quality of life [3]. Diagnosis of FMS involves the evaluation of the widespread pain index and symptom severity scale, alongside persistent symptoms such as fatigue, sleep disturbances, and cognitive impairments [1]. The treatment of FMS requires a multidisciplinary approach and often involves a combination of pharmacological and non-pharmacological strategies. However, the efficacy of these treatments varies from patient to patient, and complete resolution of symptoms is often not achievable [4]. Consequently, there is a growing interest in complementary therapies. This interest is driven by factors such as limited access to treatments, concerns about drug side effects, and persistent symptoms despite conventional therapy [5].

Ozone (O₃) is a molecule consisting of three oxygen atoms with potent oxidative activity. Its medical use dates back to the late 19th century, and in recent years, ozone therapy has garnered increasing interest, particularly in the field of complementary medicine [6]. Known for its antioxidant, anti-inflammatory, and immunomodulatory properties [7], ozone can be administered effectively to target areas through various methods, including subcutaneous, intramuscular, intra-articular, and intradiscal applications. Additionally, ozone therapy can be delivered through vaginal, rectal, or urethral insufflation and major or minor autohemotherapy [8]. As a soluble gas, ozone interacts extensively with tissue components upon contact with organic fluids such as plasma, lymph, urine, and saliva. This interaction results in effects such as reducing oxidative stress, controlling inflammation, and modulating the immune system. Ozone therapy influences the immune system by shifting macrophages from a pro-inflammatory M1 phenotype to an anti-inflammatory M2 phenotype, promoting tissue repair and homeostasis. Furthermore, it modulates inflammatory processes through the inhibition of the NLRP3 inflammasome, a key player in chronic inflammation [9]. These properties make ozone therapy a promising approach in managing various diseases. In fibromyalgia, the role of inflammatory pathways mediated by cytokines and oxidative stress offers a potential therapeutic target [10]. Ozone therapy, with its antioxidant and immunomodulatory effects, may provide relief by addressing these underlying mechanisms. However, there is limited research evaluating the efficacy of ozone therapy in FMS treatment. While existing studies report positive outcomes, further evidence is required to determine its medium- and long-term efficacy [11, 12].

The aim of this study is to retrospectively analyze the short- and medium-term efficacy of ozone therapy in patients with FMS by evaluating changes in key symptoms, including pain, quality of life, functional status, sleep quality, fatigue, anxiety, and depression. By assessing the potential of ozone therapy in alleviating FMS symptoms, this study intends to contribute to understanding its role within emerging therapeutic approaches.

## Method

### Study design

This retrospective study evaluated 25 patients diagnosed with FMS who received ozone therapy at a Physical Medicine and Rehabilitation outpatient clinic between January 2021 and March 2023. In our hospital, ozone therapy is also used for other indications in different departments and with different methods (e.g., bagging, trigger point injection, intradiscal injection); however, in our clinic, major ozone therapy is exclusively applied to FMS patients. Eligibility criteria included patients over 18 years of age who met the 2010 American College of Rheumatology (ACR) criteria for the diagnosis of FMS [1]. Patients with chronic illnesses other than FMS, history of steroid use, cancer, recent surgeries, or pregnancy were excluded. During the study period, patients were not prescribed new medications but were allowed to continue their existing treatments. Written informed consent was obtained from all participants. Ethical approval was obtained from an independent Clinical Research Ethics Committee on May 15, 2023 ( E-10840098-772.02-3001). The study was conducted in accordance with the principles of the Declaration of Helsinki. The minimum sample size was calculated using GPower 3.1 software to detect differences across three time points for primary outcomes. Although Generalized Estimating Equations (GEE) were used for analysis, the sample size calculation was based on repeated measures assumptions with an effect size of 0.50, 80% power, and 5% alpha. The required sample size was determined to be 25 patients.

### Data collection

The patients’ gender, age, height, weight, disease duration, comorbidities, smoking status, and previous treatments for FMS were retrospectively collected from patient records. According to the standard clinical protocol in our clinic, before treatment, the patients’ widespread body pain levels were assessed using a 10-cm Visual Analog Scale (VAS), and tender points were identified through physical examination and recorded. For clinical evaluation, the Fibromyalgia Impact Questionnaire (FIQ), the Hospital Anxiety and Depression Scale (HADS), the Pittsburgh Sleep Quality Index (PSQI), and the Fatigue Severity Scale (FSS) were routinely administered. These assessments were performed prior to treatment, at the end of treatment, and at the sixth month following the completion of treatment. All data were obtained from the systematically recorded patient files, and the assessments had been conducted by Physical Medicine and Rehabilitation physicians certified in ozone therapy as part of routine clinical practice.

### Measurement Scales

1. FIQ: The Turkish version of the FIQ was used to evaluate quality of life and functional status. The FIQ assesses ten domains, including physical functioning, feeling good, missed workdays, work difficulty, pain intensity, fatigue, morning tiredness, stiffness, anxiety, and depression. Except for the domain measuring “feeling good,” lower scores indicate improvement or reduced distress. FIQ is completed by the patient and has a maximum score of 100 [13].
2. HADS: This 14-item scale designed to evaluate mood disorders, anxiety, and depression symptoms. The scale includes 7 items addressing anxiety symptoms and 7 items addressing depression symptoms. Responses to each item are scored on a four-point Likert scale ranging from 0 to 3. The total score of the odd-numbered items constitutes the Anxiety subscale score (HADS-A), while the total score of the even-numbered items constitutes the Depression subscale score (HADS-D). The minimum possible score for each subscale is 0, and the maximum is 21. The validity and reliability study of the Turkish version of the scale was conducted by Aydemir [14].
3. PSQI: The PSQI has been used to assess the quality and disturbances of sleep over the past month in patients with FMS. The scale was adapted into Turkish by Ağargün et al. The PSQI includes 18 scored items, which are categorized into seven components: Subjective Sleep Quality, Sleep Latency, Sleep Duration, Habitual Sleep Efficiency, Sleep Disturbances, Use of Sleeping Medication, and Daytime Dysfunction. Each component is rated on a scale from 0 to 3. The sum of the scores for the seven components yields the total PSQI score, which ranges from 0 to 21 [15].
4. FSS: The Turkish version of the FSS, validated for reliability, was used to assess fatigue. This nine-item self-report scale scores each item from 1 (strongly disagree) to 7 (strongly agree). The total fatigue score is calculated as the mean of all items, with lower scores indicating less fatigue [16].

### Treatment protocol

Patients included in our study underwent major ozone autohemotherapy twice a week for a total of 10 sessions. The treatment protocol of twice-weekly sessions for a total of 10 sessions was chosen based on the standardized criteria from the Italian Scientific Society of Oxygen–Ozone Therapy (SIOOT). SIOOT protocols recommend at least two weekly sessions of major autohemotherapy to optimize therapeutic efficacy, as demonstrated in recent studies highlighting significant improvements in pain, fatigue, and inflammation-related symptoms in chronic conditions [17].The required materials for major ozone autohemotherapy included a vacuum-sealed glass bottle containing citrate to prevent blood clotting, a Terumo butterfly needle with 19/21 G stainless steel tips and ozone-resistant tubing, a blood transfusion set specifically designed for ozone therapy, an ozone-resistant ozone transfer set with a bacterial filter, and a 50 cc siliconized ozone syringe. The ozone gas was generated using the Turkozone Blue S (Ozon Health Services Co. Ltd. Istanbul, Turkey) ozone generator in this study. Initially, the locks on the blood transfusion and ozone transfer sets were closed and connected to the vacuum-sealed glass bottle. Venous access was then established in the patient using the butterfly needle. Once blood flow was confirmed, the tubing of the venous line was connected to the transfusion set, and the lock was opened to allow blood to flow into the bottle. After 50 cc of blood was collected, 50 mL of ozone gas at a concentration of 10–30 µg/mL was slowly introduced into the bottle via the ozone transfer set using the ozone syringe. Once the blood volume in the bottle reached 100 cc, an additional 50 mL of ozone gas at the same concentration was slowly introduced into the bottle. The blood was mixed with ozone for 20–30 s, after which the ozonated blood was reintroduced into the patient’s circulatory system through the same venous access over a period of 10–15 min. The concentration of ozone administered ranged between 10 µg/mL and 30 µg/mL, as determined by the guidelines of the International Scientific Committee of Ozone Therapy, and was adjusted before each session based on the patient’s symptoms [8].

### Statistical methods

The behavior of quantitative variables was expressed as mean ± standard deviation (SD) as a measure of central tendency. Generalized Estimating Equations (GEE) analysis was used to examine the temporal changes in repeated measurements from the same individual with respect to certain categorical and continuous variables. GEE provides the capability to perform statistical analysis by accounting for the correlation between repeated measurements. A significance level of *p* < 0.05 was set for all statistical analyses. All analyses were performed using IBM SPSS Statistics (Version 21.0 for Windows, Armonk, NY, IBM Corp.).

## Results

A total of 25 FMS patients were included in the study, with 80% (*n* = 20) being female and 20% (*n* = 5) male. The mean age of the patients was 33.08 ± 8.28 years (range: 19–45), and the mean disease duration was 29.16 ± 16.23 months (range: 8–59). The baseline demographic and clinical characteristics of the patients are summarized in Table 1.

**Table 1:** Baseline demographic and clinical characteristics of fibromyalgia patients

Parameter	Category	*n* (%) or Mean ± SD	Median (Min-Max)
Age (years)		33.08 ± 8.28	33 (19–45)
BMI (kg/m²)		27.73 ± 5.79	25.34 (19.33–39.61)
Disease Duration (months)		29.16 ± 16.23	19 (8–59)
Gender	Male	5 (20.0%)	
	Female	20 (80.0%)	
Smoking Status	Yes	10 (40.0%)	
	No	15 (60.0%)	

*SD* standard deviation; *Min-Max* minimum-maximum; *BMI* body mass index (kg/m^2^)

### Pain levels

Pain scores measured by VAS were 6.4 (95% CI: 5.65–7.15) at baseline, which decreased to 3.68 (95% CI: 3.23–4.13) at the end of the treatment (*p* < 0.001). Six months post-treatment, the pain score incerased to 4.12 (95% CI: 3.66–4.58) but remained significantly lower than baseline values (*p* < 0.001). The difference between at the end of the treatment and six months post-treatment scores was also statistically significant (*p* = 0.01) (Table 2).

**Table 2:** Comparative analysis of treatment effects at different time points

Dependent Variable	Independent Variable	Adjusted Means (95% CI)	Pairwise Comparison	*p*-value
VAS	Time (0)	6.4 (5.65, 7.15)	t (0) vs. t (2)	<0.001
	Time (2)	3.68 (3.23, 4.13)	t (0) vs. t (6)	<0.001
	Time (6)	4.12 (3.66, 4.58)	t (2) vs. t (6)	0.011
FIQ	Time (0)	59.2 (54.61, 63.79)	t (0) vs. t (2)	<0.001
	Time (2)	39.08 (33.78, 44.38)	t (0) vs. t (6)	<0.001
	Time (6)	40.12 (35.71, 44.53)	t (2) vs. t (6)	0.328
HADS-A	Time (0)	11.2 (9.03, 13.37)	t (0) vs. t (2)	<0.001
	Time (2)	3.84 (3.00, 4.68)	t (0) vs. t (6)	<0.001
	Time (6)	6.92 (4.96, 8.88)	t (2) vs. t (6)	0.003
HADS-D	Time (0)	10.36 (8.37, 12.35)	t (0) vs. t (2)	<0.001
	Time (2)	3.36 (2.73, 3.99)	t (0) vs. t (6)	<0.001
	Time (6)	5.8 (4.41, 7.19)	t (2) vs. t (6)	0.003
PSQI	Time (0)	11.2 (9.03, 13.37)	t (0) vs. t (2)	<0.001
	Time (2)	6.36 (5.08, 7.64)	t (0) vs. t (6)	<0.001
	Time (6)	8.84 (6.82, 10.86)	t (2) vs. t (6)	0.004
FSS	Time (0)	5.48 (5.04, 5.92)	t (0) vs. t (2)	<0.001
	Time (2)	3.24 (2.7, 3.78)	t (0) vs. t (6)	0.052
	Time (6)	4.56 (3.88, 5.24)	t (2) vs. t (6)	0.002
Tender Points	Time (0)	14.36 (13.4, 15.32)	t (0) vs. t (2)	<0.001
	Time (2)	9.8 (8.63, 10.97)	t (0) vs. t (6)	<0.001
	Time (6)	10.12 (8.78, 11.46)	t (2) vs. t (6)	0.289

*VAS* visual analog scale; *FIQ* fibromyalgia impact questionnaire; *HADS-A* hospital anxiety and depression scale-anxiety score; *HADS-D* hospital anxiety and depression scale-depression score; *PSQI* Pittsburgh sleep quality index; *FSS* fatigue severity scale; *Time (0)* prior to treatment; *Time (2)* at the end of treatment; *Time (6)* at the sixth month following the completion of treatment; *CI* confidence intervals

### Functional disability

The functional disability score, which was 59.2 (95% CI: 54.61–63.79) at baseline, decreased to 39.08 (95% CI: 33.78–44.38) at the end of the treatment (*p* < 0.001). At six months post-treatment, the score increased to 40.12 (95% CI: 35.71–44.53). However, the change between at the end of the treatment and six-month scores was not statistically significant (*p* = 0.328). The scores end of the treatment and six months post-treatment were found to be statistically significantly lower compared to baseline (*p* < 0.001). (Table 2).

### Anxiety and depression

Anxiety scores decreased from 11.2 (95% CI: 9.03–13.37) at baseline to 3.84 (95% CI: 3.00–4.68) at the end of the treatment (*p* < 0.001). At six months post-treatment, the anxiety score increased to 6.92 (95% CI: 4.96–8.88). The anxiety scores after treatment and 6 months post-treatment were found to be statistically significantly lower compared to baseline.(*p* < 0.001) However, the scores at six months post-treatment were found to be statistically significantly higher compared to the post-treatment values (*p* = 0.003). Depression scores showed a similar trend, dropping from 10.36 (95% CI: 8.37–12.35) at baseline to 3.36 (95% CI: 2.73–3.99) at the end of the treatment (*p* < 0.001). At six months post-treatment, the depression score rose to 5.8 (95% CI: 4.41–7.19), and this increase was also statistically significant (*p* = 0.003) (Table 2).

### Sleep quality

The sleep quality score, which was initially 11.2 (95% CI: 9.03–13.37), decreased to 6.36 (95% CI: 5.08–7.64) at the end of the treatment (*p* < 0.001). Six months post-treatment, this score increased to 8.84 (95% CI: 6.82–10.86) but remained significantly lower compared to baseline (*p* = 0.004) (Table 2).

### Fatigue

The baseline fatigue score of 5.48 (95% CI: 5.04–5.92) decreased to 3.24 (95% CI: 2.7–3.78) at the end of the treatment (*p* < 0.001). Six months post-treatment, the fatigue score rose to 4.56 (95% CI: 3.88–5.24) but remained significantly lower than the baseline level (*p* = 0.002) (Table 2).

### Tender points

The number of tender points, initially 14.36 (95% CI: 13.4–15.32), decreased to 9.8 (95% CI: 8.63–10.97) at the end of the treatment (*p* < 0.001). Six months post-treatment, the number of tender points increased to 10.12 (95% CI: 8.78–11.46), but this increase was not found to be statistically significant (*p* = 0.289). The number of tender points six months post-treatment was found to be statistically significantly lower compared to the number of tender points at baseline (*p* < 0.001). (Table 2).

## Discussion

This retrospective study evaluated the acute and mid-term efficacy of ozone therapy in patients diagnosed with FMS by examining changes in major symptoms such as pain, quality of life, functional status, sleep quality, fatigue, anxiety, and depression. In our study, comparisons of pre-treatment, post-treatment, and six-month follow-up results revealed that ozone therapy provided significant improvements in symptoms and quality of life, supporting its effectiveness as a complementary approach in the management of fibromyalgia.

Ozone therapy has been gaining increasing attention as a complementary treatment modality for musculoskeletal disorders due to its biological effects. It is known that ozone interacts with biological components to induce moderate oxidative stress, thereby exhibiting antioxidant, anti-inflammatory, and immunomodulatory properties [6, 8, 18, 19]. Additionally, it has been reported to regulate the expression of genes associated with inflammation and oxidative stress by activating intracellular signaling pathways and to reduce pain perception through the increased release of serotonin and endogenous opioids [8]. A study by Moreno-Fernández et al. objectively demonstrated that ozone autohemotherapy significantly increased serum serotonin levels and decreased oxidative stress markers, such as malondialdehyde and protein carbonyl levels, in fibromyalgia patients [20]. Ozone, which can be administered through various methods (such as intra-articular, paravertebral injections, or rectal insufflation), has been shown to have the potential to alleviate symptoms and support functional improvement in conditions such as low back pain, knee osteoarthritis, tendinopathies, and fibromyalgia [21–24]. Ozone therapy, which can be safely applied at low doses, is considered a complementary option for mitigating symptoms of chronic conditions like fibromyalgia due to its effects on managing oxidative stress and inflammation [2]. However, it is important to note that high doses of ozone may induce toxic effects, and treatment protocols should be optimized accordingly [8].

In a prospective randomized clinical trial conducted by Eldemrdash et al., the short-term efficacy of ozone, betamethasone, and their combined application was evaluated in patients with chronic musculoskeletal pain [25]. In this study, both ozone and betamethasone treatments were administered as trigger point injections. Assessments conducted at 3 days, 1 week, and 3 weeks post-treatment revealed that ozone therapy significantly and rapidly reduced pain. Furthermore, it was reported that ozone therapy exhibited antioxidant effects by increasing the reduced/oxidized glutathione ratio and improved mitochondrial function by enhancing mitochondrial copy number. However, as the study’s evaluation period was limited to 3 weeks, it did not provide information on the medium- and long-term effects of the treatment. Similarly, in our study, ozone therapy was found to improve symptoms such as pain, sleep quality, and fatigue in patients with fibromyalgia. However, follow-up results up to the 6th month indicated a partial decrease in treatment efficacy. These findings suggest that ozone therapy is not only effective in pain reduction but also in managing oxidative stress, highlighting the need for further research to evaluate its long-term efficacy.

Musculoskeletal disorders and associated conditions such as sleep disturbances, pain, depression, and fatigue are significant clinical challenges that require a multidisciplinary approach. A randomized controlled trial conducted in China evaluated the effects of ozone autohemotherapy, in addition to pharmacological treatment, on sleep quality, pain (VAS score), depression, and fatigue in patients with insomnia and myofascial pain syndrome over a 6-month follow-up period [26]. This study demonstrated that ozone autohemotherapy provided superior symptom improvement and significantly enhanced quality of life compared to pharmacological treatment alone. The antioxidant effects of ozone, which help control inflammation and improve oxygen delivery, played a crucial role in achieving these outcomes. Similarly, in our study, ozone therapy was observed to provide significant short- and medium-term improvements in symptoms such as pain, fatigue, sleep quality, and depression in patients with FMS. However, partial symptom recurrence was noted at the 6-month follow-up. These findings indicate that ozone therapy is an effective short- and medium-term treatment option and should be considered as a complementary approach in managing sleep disorders and chronic pain syndromes in clinical practice.

Ozone therapy in FMS can be administered through various methods and frequencies, allowing for optimization tailored to individual needs. In a study conducted by Tirelli et al., ozone therapy was applied to 65 fibromyalgia patients using autohemotransfusion and rectal insufflation methods. The treatment involved twice-weekly sessions during the first month, followed by bi-weekly maintenance therapy sessions [21]. The outcomes were monitored through regular evaluations during the treatment period, focusing on short-term effects. It was observed that over 70% of the patients achieved more than 50% improvement in their symptoms. In contrast, our study differed by exclusively evaluating a 10-session protocol of major ozone autohemotherapy and analyzing its medium-term efficacy through a 6-month follow-up period. When these two studies are considered together, ozone therapy appears to be a promising complementary treatment option for managing fibromyalgia symptoms effectively in both the short and medium term.

This study has certain limitations. Our research is based on a retrospective design, relying on data obtained from previously collected records. This makes it challenging to establish causality and may hinder the full explanation of the effects of certain factors on the outcomes. Additionally, our study exclusively evaluated the major ozone autohemotherapy method, without considering other systemic ozone application methods such as rectal insufflation. Furthermore, the absence of a control group limits the ability to comparatively assess treatment efficacy. Additionally, the lack of a case-control design with non-fibromyalgia patients makes it more difficult to distinguish the specific effects of ozone therapy from placebo and psychological influences. The use of subjective assessment scales also introduces the potential for results to be influenced by individual perception, which may compromise the objectivity of the evaluations.

In conclusion, this study demonstrated the positive effects of ozone therapy on symptoms such as pain, quality of life, functional status, sleep quality, fatigue, anxiety, and depression in patients with FMS. The six-month follow-up period provided a significant advantage in assessing the medium-term efficacy of the treatment. A comparison of at the end of the treatment results with those at the six-month follow-up revealed partial relapses in symptoms such as pain, sleep quality, fatigue, anxiety, and depression. This finding suggests that additional sessions or maintenance protocols may be required to sustain treatment efficacy. Accordingly, to maintain treatment efficacy after the sixth month, ozone therapy sessions at regular intervals or individualized maintenance protocols can be implemented based on the patients’ symptom levels. In the future, prospective studies with larger sample sizes, control groups, and evaluations of different ozone application methods and long-term outcomes are needed. Incorporating objective measurement methods, such as biochemical parameters, into these studies would also be beneficial. Such research will help clarify the role of ozone therapy in the management of FMS more definitively.

## Electronic supplementary material

Below is the link to the electronic supplementary material.

Supplementary Material 1

Supplementary Material 2

Supplementary Material 3
