# Supplementary material for: Short- and Medium-Term effects of major Ozone therapy on disease parameters in fibromyalgia syndrome: A retrospective study
Source: Rheumatol Int. 2025 Mar 12;45(4):72. doi: 10.1007/s00296-025-05827-1 (PMC11903636; doi:10.1007/s00296-025-05827-1)
Supplement: Supplementary file 1 — Supplementary Material 1 [file 296_2025_5827_MOESM1_ESM.docx]

FİBROMİYALJİ HASTALARI OZON TERAPİ TAKİP FORMU

Değerlendirme tarihi:

Ad-soyad:

Yaş:

Boy: Kilo: BMI:

Ek hastalık:

Sigara kullanımı:

İlaç kullanımı: nsaid: kas gevşetici: anti-depresan: diğer:

Fibromiyalji süresi:

Düzenli egzersiz alışkanlığı:

Lokal tedavi gerekliliği:

OZON TERAPİ DOZLAR:

| 1.DOZ | 2.DOZ | 3.DOZ | 4.DOZ | 5.DOZ | 6.DOZ | 7.DOZ | 8.DOZ | 9.DOZ | 10.DOZ |
| --- | --- | --- | --- | --- | --- | --- | --- | --- | --- |
|  |  |  |  |  |  |  |  |  |  |

|  | TEDAVİ ÖNCESİ | TEDAVİ SONRASI  1.AY | TEDAVİ SONRASI  6. AY |
| --- | --- | --- | --- |
| HASSAS NOKTA SAYISI |  |  |  |
| GENEL AĞRI VAS |  |  |  |
| FIQ |  |  |  |
| HAD-A  HAD-D  HAD-TOTAL |  |  |  |
| FSS |  |  |  |
| PSQI |  |  |  |
