# Supplementary material for: Short- and Medium-Term effects of major Ozone therapy on disease parameters in fibromyalgia syndrome: A retrospective study
Source: Rheumatol Int. 2025 Mar 12;45(4):72. doi: 10.1007/s00296-025-05827-1 (PMC11903636; doi:10.1007/s00296-025-05827-1)
Supplement: Supplementary file 2 — Supplementary Material 2 [file 296_2025_5827_MOESM2_ESM.pdf]

T.C.
İSTANBUL MEDİPOL ÜNİVERSİTESİ
Girişimsel Olmayan Klinik Araştırmalar Etik Kurulu Başkanlığı

Sayı : E-10840098-772.02-3001 15/05/2023

Konu: Etik Kurulu Kararı

Sayın Ahmet ÜŞEN

Üniversitemiz Girişimsel Olmayan Klinik Araştırmalar Etik Kuruluna yapmış olduğunuz "Fibromiyalji Sendromunda Major Ozon Tedavisinin Hastalık Parametreleri üzerine etkisi: Retrospektif Bir Çalışma" isimli başvurunuz etik kurulu toplantımızda değerlendirilerek uygun görülmüş olup Etik Kurulu kararı ekte sunulmuştur.

Bilgilerinize rica ederim.

Dr. Öğr. Üyesi Mahmut TOKAÇ
Girişimsel Olmayan Klinik Araştırmalar
Etik Kurulu Başkanı

Ek
-Karar Formu (2 sayfa)

Bu belge, güvenli elektronik imza ile imzalanmıştır.
Evrağınızı https://turkiye.gov.tr/istanbul-medipol-universitesi-ebys linkinden C60C702BX8 kodu ile dogrulayabilirsiniz.

BAŞVURU BİLGİLERİ		
	**ARAŞTIRMANIN AÇIK ADI**	Fibromiyalji Sendromunda Major Ozon Tedavisinin Hastalık Parametreleri üzerine etkisi: Retrospektif Bir Çalışma
	**KOORDİNATÖR/SORUMLU ARAŞTIRMACI UNVANI/ADI/SOYADI**	Ahmet ÜŞEN
	**KOORDİNATÖR/SORUMLU ARAŞTIRMACININ UZMANLIK ALANI**	Doktor Öğretim Üyesi / Fiziksel Tıp ve Rehabilitasyon
	**KOORDİNATÖR/SORUMLU ARAŞTIRMACININ BULUNDUĞU MERKEZ**	İstanbul
	**DESTEKLEYİCİ**	-
	**ARAŞTIRMAYA KATILAN MERKEZLER**	TEK MERKEZ ☒ ÇOK MERKEZ ☐ ULUSAL ☒ ULUSLARARASI ☐

Değerlendirilen Belgeler	**Belge Adı**	**Tarihi**	**Versiyon Numarası**	**Dili**
	ARAŞTIRMA PROTOKOLÜ/PLANI			Türkçe ☐ İngilizce ☐ Diğer ☐
	OLGU RAPOR FORMU			Türkçe ☐ İngilizce ☐ Diğer ☐
	BİLGİLENDİRİLMİŞ GÖNÜLLÜ OLUR FORMU	06.05.2023	V1.0	Türkçe ☒ İngilizce ☐ Diğer ☐

Karar Bilgileri		
	**Karar No:454**	**Tarih: 11.05.2023**
	Yukarıda bilgileri verilen Girişimsel Olmayan Klinik Araştırmalar Etik Kurulu başvuru dosyası ile ilgili belgeler araştırmanın gerekçe, amaç, yaklaşım ve yöntemleri dikkate alınarak incelenmiş ve araştırmanın etik ve bilimsel yönden uygun olduğuna **“oybirliği”** ile karar verilmiştir.	

Bu belge, güvenli elektronik imza ile imzalanmıştır.
Evrağınızı https://turkiye.gov.tr/istanbul-medipol-universitesi-ebys linkinden C60C702BX8 kodu ile dogrulayabilirsiniz.

İSTANBUL MEDİPOL ÜNİVERSİTESİ GİRİŞİMSEL OLMAYAN KLİNİK ARAŞTIRMALAR ETİK KURULU	
**BAŞKANIN UNVANI / ADI / SOYADI**	Dr. Öğr. Üyesi Mahmut TOKAÇ

Unvanı/Adı/Soyadı	Uzmanlık Alanı	Kurumu	Cinsiyet		Araştırma ile ilişki		Katılım *		İmza
Dr. Öğr. Üyesi Mahmut TOKAÇ	Tıp Tarihi ve Etik	İstanbul Medipol Üniversitesi	E ☒	K ☐	E ☐	H ☒	E ☒	H ☐	E imzalıdır
Prof. Dr. Mete ÜNGÖR	Endodonti	İstanbul Medipol Üniversitesi	E ☒	K ☐	E ☐	H ☒	E ☒	H ☐	E imzalıdır
Doç. Dr. Mehmet Kemal ÖZDEMİR	Elektrik ve Elektronik	İstanbul Medipol Üniversitesi	E ☒	K ☐	E ☐	H ☒	E ☒	H ☐	E imzalıdır
Doç. Dr. İlknur KESKİN	Histoloji ve Embriyoloji	İstanbul Medipol Üniversitesi	E ☐	K ☒	E ☐	H ☒	E ☒	H ☐	E imzalıdır
Doç. Dr. Devrim TARAKCI	Fizyoterapi ve Rehabilitasyon	İstanbul Medipol Üniversitesi	E ☒	K ☐	E ☐	H ☒	E ☒	H ☐	E imzalıdır
Dr. Öğr. Üyesi Neziha HACIHASANOĞLU ÇAKMAK	Biyokimya	İstanbul Medipol Üniversitesi	E ☐	K ☒	E ☐	H ☒	E ☒	H ☐	E imzalıdır
Dr. Öğr. Üyesi Pakize YİĞİT	Sayısal Yöntemler/ Biyoistatistik	İstanbul Medipol Üniversitesi	E ☐	K ☒	E ☐	H ☒	E ☐	H ☒	Katılmadı

* :Toplantıda Bulunma

Bu belge, güvenli elektronik imza ile imzalanmıştır.
Evrağınızı https://turkiye.gov.tr/istanbul-medipol-universitesi-ebys linkinden C60C702BX8 kodu ile dogrulayabilirsiniz.
