# Supplementary material for: Short- and Medium-Term effects of major Ozone therapy on disease parameters in fibromyalgia syndrome: A retrospective study
Source: Rheumatol Int. 2025 Mar 12;45(4):72. doi: 10.1007/s00296-025-05827-1 (PMC11903636; doi:10.1007/s00296-025-05827-1)
Supplement: Supplementary file 3 — Supplementary Material 3 [file 296_2025_5827_MOESM3_ESM.docx]

# **Table 1. Baseline Demographic and Clinical Characteristics of Fibromyalgia Patients**

| **Parameter** | **Category** | **n (%) or Mean ± SD** | **Median (Min-Max)** |
| --- | --- | --- | --- |
| **Age (years)** |  | 33.08 ± 8.28 | 33 (19 - 45) |
| **BMI (kg/m²)** |  | 27.73 ± 5.79 | 25.34 (19.33 - 39.61) |
| **Disease Duration (months)** |  | 29.16 ± 16.23 | 19 (8 - 59) |
| **Gender** | Male | 5 (20.0%) |  |
|  | Female | 20 (80.0%) |  |
| **Smoking Status** | Yes | 10 (40.0%) |  |
|  | No | 15 (60.0%) |  |

**Table 1:** Standard Deviation: SD, Min-Max: Minimum-Maximum; BMI: Body Mass Index (kg/m²)

# **Table 2. Comparative Analysis of Treatment Effects at Different Time Points**

| **Dependent Variable** | **Independent Variable** | **Adjusted Means (95% CI)** | **Pairwise Comparison** | **p-value** |
| --- | --- | --- | --- | --- |
| **VAS** | Time (0) | 6.4 (5.65, 7.15) | t (0) vs. t (2) | <0.001 |
|  | Time (2) | 3.68 (3.23, 4.13) | t (0) vs. t (6) | <0.001 |
|  | Time (6) | 4.12 (3.66, 4.58) | t (2) vs. t (6) | 0.011 |
| **FIQ** | Time (0) | 59.2 (54.61, 63.79) | t (0) vs. t (2) | <0.001 |
|  | Time (2) | 39.08 (33.78, 44.38) | t (0) vs. t (6) | <0.001 |
|  | Time (6) | 40.12 (35.71, 44.53) | t (2) vs. t (6) | 0.328 |
| **HADA** | Time (0) | 11.2 (9.03, 13.37) | t (0) vs. t (2) | <0.001 |
|  | Time (2) | 3.84 (3.00, 4.68) | t (0) vs. t (6) | <0.001 |
|  | Time (6) | 6.92 (4.96, 8.88) | t (2) vs. t (6) | 0.003 |
| **HADD** | Time (0) | 10.36 (8.37, 12.35) | t (0) vs. t (2) | <0.001 |
|  | Time (2) | 3.36 (2.73, 3.99) | t (0) vs. t (6) | <0.001 |
|  | Time (6) | 5.8 (4.41, 7.19) | t (2) vs. t (6) | 0.003 |
| **PSQI** | Time (0) | 11.2 (9.03, 13.37) | t (0) vs. t (2) | <0.001 |
|  | Time (2) | 6.36 (5.08, 7.64) | t (0) vs. t (6) | <0.001 |
|  | Time (6) | 8.84 (6.82, 10.86) | t (2) vs. t (6) | 0.004 |
| **FSS** | Time (0) | 5.48 (5.04, 5.92) | t (0) vs. t (2) | <0.001 |
|  | Time (2) | 3.24 (2.7, 3.78) | t (0) vs. t (6) | 0.052 |
|  | Time (6) | 4.56 (3.88, 5.24) | t (2) vs. t (6) | 0.002 |
| **Tender Points** | Time (0) | 14.36 (13.4, 15.32) | t (0) vs. t (2) | <0.001 |
|  | Time (2) | 9.8 (8.63, 10.97) | t (0) vs. t (6) | <0.001 |
|  | Time (6) | 10.12 (8.78, 11.46) | t (2) vs. t (6) | 0.289 |

### **Table 2:** VAS: Visual Analog Scale; FIQ: Fibromyalgia Impact Questionnaire; HADA: Hospital Anxiety Scale; HADD: Hospital Depression Scale; PSQI: Pittsburgh Sleep Quality Index; FSS: Fatigue Severity Scale. Time (0): Prior to treatment; Time (2): At the end of treatment; Time (6): At the sixth month following the completion of treatment; CI: Confidence Intervals
